# Retracted: Evidences of Protective Potentials of Microdoses of Ultra-High Diluted Arsenic Trioxide in Mice Receiving Repeated Injections of Arsenic Trioxide

**DOI:** 10.1155/2021/5782851

**Published:** 2021-01-11

**Authors:** Evidence-Based Complementary and Alternative Medicine

*Evidence-Based Complementary and Alternative Medicine* has retracted the article titled “Evidences of Protective Potentials of Microdoses of Ultra-High Diluted Arsenic Trioxide in Mice Receiving Repeated Injections of Arsenic Trioxide” [1], due to concerns with the figures as originally raised on PubPeer [2].

A number of overlapping regions were identified between Figures 1(d) and 1(f). The authors explained that some overlapping areas of histological features are to be expected; however, this was not determined to be a satisfactory explanation. Additionally, undeclared gel splicing was identified in Figure 3. The article is therefore being retracted with the agreement of the editorial board. The authors do not agree to the retraction.
